# Development of drugs for celiac disease: review of endpoints for Phase 2 and 3 trials

**DOI:** 10.1093/gastro/gov006

**Published:** 2015-02-26

**Authors:** Klaus Gottlieb, Jill Dawson, Fez Hussain, Joseph A. Murray

**Affiliations:** ^1^Immunology and Internal Medicine - Medical Strategy & Science, Quintiles, Durham, NC, USA, ^2^Corporate Communications, Quintiles, Durham, NC, USA and ^3^Division of Gastroenterology and Hepatology, Mayo Clinic, Rochester, MN, USA

**Keywords:** celiac disease, clinical trials, endpoints

## Abstract

Celiac disease is a lifelong disorder for which there is currently only one known, effective treatment: a gluten-free diet. New treatment approaches have recently emerged; several drugs are in Phase 2 trials and results appear promising; however, discussion around regulatory endpoints is in its infancy. We will briefly discuss the drugs that are under development and then shift our attention to potential trial endpoints, such as patient-reported outcomes, histology, serology, gene expression analysis and other tests. We will outline the differing requirements for proof-of-concept Phase 2 trials and Phase 3 registration trials, with a particular emphasis on current thinking in regulatory agencies. We conclude our paper with recommendations and a glossary of regulatory terms, to enable readers who are less familiar with regulatory language to take maximum advantage of this review.

## Introduction

Research into therapy for celiac disease is currently at a particularly interesting point. There are now several different drugs in development but no agreement exists on the best endpoints for registration trials. We will review drugs in the ‘pipeline’, examine the experience to date, and discuss possible regulatory endpoints in detail.

## Pathology and incidence of celiac disease

Celiac disease is an immune-mediated small intestinal enteropathy, triggered by the ingestion of gluten in the genetically susceptible, which results in villous atrophy. The presenting symptoms may range from diarrhea, constipation, vomiting, malnutrition, or failure to thrive, to chronic fatigue, joint pain, anemia, osteoporosis, or migraines [1]. The prevalence of celiac disease has increased over the past 50 years and the rate of diagnosis has risen over the past two decades [2].

Celiac disease affects some two million Americans, of whom around 83% are not diagnosed [3, 4], and 3.5 million Europeans [5]. This makes celiac disease one of the most common food-related, lifelong disorders worldwide [6]. Although many individuals with this disease remain undiagnosed, others who have been given the diagnosis do not actually have the disease; additionally, there is an increasing number of people who have no diagnosis of celiac disease but who nevertheless adhere to a gluten-free diet (GFD). Rubio-Tapia *et al.* reported that, among the general population of the USA that has not been diagnosed with celiac disease, the prevalence of gluten exclusion was similar to that of actual celiac disease [7]. The environmental trigger (gluten derived from wheat, rye and barley), the genetic predisposition conferred by the human leukocyte antigen (HLA)-DQ2 and HLA-DQ8 haplotypes, and many steps in the disease pathogenesis are known. Novel alternative treatments or adjunctive therapies to a gluten-free diet—which is currently the only available and effective treatment for the condition—are increasingly being suggested [8].

This paper covers celiac disease *per se*, which is the best-characterized of the spectrum of gluten-related disorders [9]. A gluten-free diet is central to the management of celiac disease and has historically been the only treatment, although several potential therapies are now under development. There is also a widespread public interest in gluten-free foods reflected, for example, in recent U.S. Food and Drug Administration (the FDA) food labeling regulations [10]. Market research by the National Purchase Diary Group (NPD) shows a gradual but steady increase in the percentage of adults who say they are cutting down on gluten or avoiding it completely—currently more than one in every four adults [11].

Celiac disease is typically detected by serological testing of celiac-specific antibodies, and the diagnosis is usually confirmed by duodenal mucosal biopsies [12]. Both serology and biopsy should be performed while the individual is following a gluten-containing diet [12]; however, potential celiac disease sufferers who have started themselves on a celiac free-diet before a work-up was completed, may be harder to diagnose. This is especially true if the patient carries the HLA-DQ2 and HLA-DQ8 haplotypes, as do 30–50% of the unaffected population [13]. In these circumstances, a new diagnostic test—currently still under development and based on cytokine release assay in response to a gluten challenge—could be helpful [14]. Although duodenal biopsies are still considered essential in adults, an alternative diagnostic strategy has been suggested, which avoids biopsies in children who have tissue transglutaminase levels higher than 10 times the upper limit of normal [15].

It is generally believed that the IgA anti-tissue transglutaminase (tTG) is the single best serological test to use for the detection of celiac disease. While celiac disease can be recognized endoscopically by visual inspection, especially if water immersion is used to enhance the detection of villi, a normal endoscopic appearance does not preclude the diagnosis [16].

At present, treatment primarily involves a GFD, an approach that demands significant patient education, motivation, and follow-up. The GFD also imposes a heavy treatment burden on patients, given the requirement for constant vigilance for gluten contamination. Not surprisingly, a large proportion of patients report inadvertent or deliberate exposure to gluten [17]. Non-responsive celiac disease is frequently reported, especially among individuals diagnosed in adulthood, and may be in part related to trace gluten contamination [18]. In many adult patients—perhaps the majority—the intestine fails to heal even after two years of the GFD [19].

## Research and development ‘pipeline’ for celiac disease drugs & continuing clinical trials

**Figure 1.**
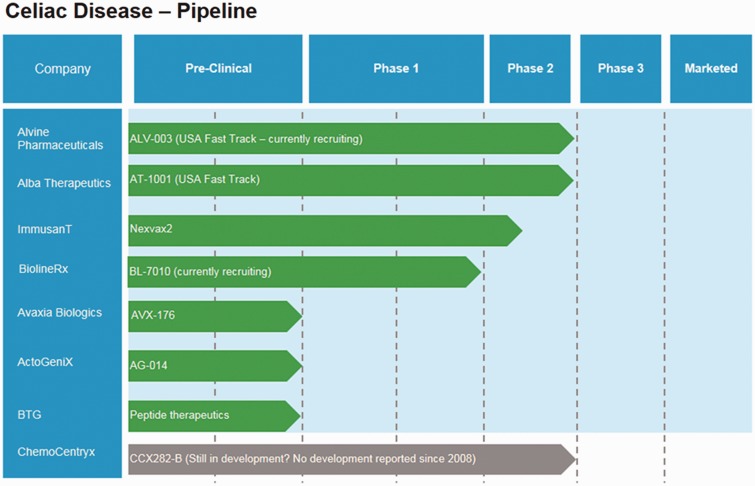
The R&D ‘pipeline’ for celiac disease (based on information from www.clinicaltrials.gov and www.clinicaltrialsregister.eu)

Clinical trials on drugs to treat celiac disease are at an early stage of evolution, with no products having reached Phase 3 to date (Figure 1). Products in development include:

- ALV003, Alvine Pharmaceuticals’ lead clinical candidate for celiac disease, which is currently being studied in Phase 2b [20]. ALV003 is an orally administered mixture of two recombinant gluten-specific proteases—a cysteine protease (EP-B2) and a prolyl endopeptidase (PEP)—which have been shown *in vitro* to degrade gluten.

In a Phase 2 study with ALV003, adults with biopsy-proven celiac disease were randomly assigned to groups receiving ALV003 (*n *=* *20) or placebo (*n *=* *21), together with a daily 2 g gluten challenge. Duodenal biopsies were collected at baseline and after the gluten challenge. The ratio of villus-height-to-crypt-depth and densities of intra-epithelial lymphocytes were the primary endpoints. Biopsies from subjects in the placebo group showed evidence of mucosal injury after gluten challenge (mean villus height-to-crypt depth ratio changed from 2.8 before challenge to 2.0 afterward; *P = *0.0007; density of CD3+ intraepithelial lymphocytes changed from 61 to 91 cells/mm after challenge; *P = *0.0003). In contrast, no significant mucosal deterioration was observed in biopsies from the ALV003 group. Between groups, morphologic changes and CD3+ intraepithelial lymphocyte counts differed significantly from baseline to Week 6 (*P = *0.0133 and *P = *0.0123, respectively). Interestingly, there were no statistically significant differences in symptoms between groups [21].

Based on the promising Phase 2a results, a Phase 2b study is now being conducted. The study is evaluating the safety and efficacy of ALV003 at different dose levels administered over a twelve-week period in 500 celiac disease patients in the USA, Canada and Europe, who are symptomatic despite attempting to follow a GFD. The primary efficacy endpoint for the study is the change in small intestinal mucosal morphology, as measured by the change in villus-height-to-crypt-depth ratio (Vh:Cd) from baseline and week 12 assessments. Secondary endpoints are the changes in density in intestinal intraepithelial lymphocytes and celiac disease-specific symptoms during the study. Other outcomes to be evaluated include changes in celiac disease serologies and quality-of-life measures [20].

Aspergillus niger prolyl endoprotease (AN-PEP) is an endopeptidase, like the PEP component of ALV003, which can break down gluten. The enzyme is active between pH 2 and pH 8, with an optimum activity at pH 4–5, and is therefore effective at the pH levels present in the stomach and small intestine [22]. AN-PEP was evaluated in a recent small, double-blind, placebo controlled, randomized trial on 16 patients with a diagnosis of celiac disease—as confirmed by positive serology—with sub-total or total villous atrophy on duodenal biopsies, who adhered to a strict GFD, resulting in normalized antibodies and mucosal healing classified as Marsh 0 or I [22]. In a randomized, double-blind, placebo-controlled pilot study, patients consumed toast (approximately 7* *g/day gluten) with AN-PEP for 2 weeks (safety phase). After a 2-week washout period with adherence to the usual GFD, 14 patients were randomized to gluten intake with either AN-PEP or placebo for 2 weeks (efficacy phase). No serious adverse events occurred and no patients withdrew during the trial. The mean score for the gastrointestinal subcategory of the celiac disease quality (CDQ) was relatively high throughout the study, indicating that AN-PEP was well tolerated. In the efficacy phase, the CDQ scores of patients consuming gluten with placebo or gluten with AN-PEP did not significantly deteriorate and, moreover, no differences between the groups were observed. The authors conclude in their discussion that “with hindsight, the study should possibly have been designed for a much longer period of time with many more patients” [22].

- Larazotide acetate (AT-1001) is Alba Therapeutics Corporation’s investigational product, a first-in-class tight junction regulator, intended for the treatment of patients with celiac disease. It has been hypothesized that celiac disease is accompanied by raised paracellular permeability, accompanied by an inflammatory cascade within the bowel, which is controlled by tight junctions. Alba has reported positive results from a double-blind, placebo-controlled, Phase 2b trial in February 2014 [23]. This evaluated the efficacy and safety of larazotide acetate in 342 celiac disease patients who had symptoms despite being on a GFD. The trial consisted of a four-week placebo run-in, 12 weeks of randomized therapy, and four weeks of post-treatment follow-up. Patients were randomized to four groups: a placebo group or larazotide 0.5, 1.0, or 2.0* *mg, three times per day. Treatment with the lowest of three doses of larazotide was associated with significant improvement in the primary outcome, i.e. the average on-treatment score in the Celiac Disease Gastrointestinal Symptom Rating Scale (CeD GSRS) domains of Diarrhea, Indigestion, and Abdominal pain. Analysis of individual components of the rating scale, proprietary to Alba Therapeutics [24], showed consistent improvement with larazotide for each parameter [25].

The Phase 2b study discussed above was preceded by a separate, dose-ranging, placebo-controlled study of 86 patients with celiac disease controlled through diet [26]. The aim of this study was to evaluate the efficacy and tolerability of larazotide in protecting against gluten-induced intestinal permeability and worsening gastrointestinal symptoms. Study participants were randomly assigned to larazotide acetate (0.25, 1, 4, or 8* *mg) or placebo three times a day, with or without gluten challenge (2.4* *g/day) for 14 days. The primary efficacy outcome, an improvement in the lactulose/mannitol (LAMA) fractional excretion ratio (an experimental biomarker for intestinal permeability) was not met; however, the 0.25 and 4.0* *mg doses of larazotide acetate showed statistically significant prevention of severe worsening of gastrointestinal symptoms.

An exploratory study published in 2013 [27] examined the effect of larazotide acetate on intestinal permeability, development of antibodies to tTG and celiac disease symptoms during a gluten challenge that exceeded the likely level of accidental gluten ingestion in individuals whose disease was well controlled by a GFD. In the larazotide acetate 1 mg group, a reduction in the expected increase was seen in the urinary LAMA ratio but the difference was not statistically significant as compared with placebo. Changes in pre-specified secondary endpoints suggest that larazotide acetate reduced antigen exposure, as shown by lowered production of anti-tTG antibodies. Larazotide acetate also reduced gastrointestinal symptoms upon gluten challenge.

- Nexvax2^®^: ImmusanT’s peptide-based therapeutic celiac disease vaccine.

According to press releases from ImmusanT [28], the therapeutic vaccine Nexvax2 combines three proprietary peptides that elicit an immune response in celiac disease patients who carry the immune recognition gene HLA-DQ2. Similarly to treatments for allergies, the vaccine is designed to reprogram gluten-specific T cells triggered by the patient’s immune response to the protein. According to ImmusanT, the objective is for Nexvax2 to restore celiac patients’ immune tolerance to gluten, reduce inflammation in the nutrient-absorbing villi that line the small intestine, return the intestine to a healthy state, and allow patients to eat a normal diet [28].

The company says that early clinical trials have so far proven promising, with Phase 1b trial results demonstrating clear proof of mechanism and Phase 2 trials expected to begin in 2015—but details are not known at the time of this writing (December 2014) [29].

- BL-7010: BiolineRx’s non-absorbable, high molecular weight polymer with a high affinity for gliadins, the immunogenic peptides present in gluten that cause celiac disease. The product acts by sequestering gliadins. Experiments *in vivo* in a murine model of celiac disease have shown that BL-7010 prevents pathological damage to the small intestine, helps to preserve the integrity of the intestinal mucosa and reduces inflammation [30]. Although the company website lists BL-7010 as being in pre-clinical development, clinicaltrials.gov shows an active recruiting Phase 1 safety study [31].
- AVX176, from Avaxia Biologics, is an investigational oral antibody drug that is the subject of U.S. composition of matter patent 8,071,101, “Antibody Therapy for Treatment of Diseases Associated with Gluten Intolerance.” The patent, which expires on May 27 2029, provides broad coverage for treating celiac disease using orally administered antibodies produced by Avaxia’s proprietary platform technology [32].
- ActoGenX is carrying out discovery research in celiac disease with its range of ActoBiotics^™^, which use *Lactococcus lactis* as an expression system to locally secrete bio-therapeutics such as cytokines, antibodies, hormones, etc. [33]. Early pre-clinical work with a genetically altered *L. lactis* secreting a peptide derived from gliadin demonstrated an *in vivo* suppression of gluten sensitization. Specifically, Huigbregtse *et al.* engineered *L. lactis* to secrete a deamidated DQ8 gliadin epitope (LL-eDQ8d) and studied the induction of Ag-specific tolerance in NOD ABo DQ8 transgenic mice [34]. Although apparently not part of the ActoGenX development program, recent work by Galipeau *et al.* also deserves mention in this context. The group treated gluten-sensitive mice with elafin, a serine protease inhibitor, delivered by the *L. lactis* vector, and found normalization of inflammation, improved permeability, and maintained ZO-1 expression. There is speculation that this is due to reduced deamidation of gliadin peptide [31].
- Chemocentryx’s CCR9 (vercirnon, which is also known as Traficet-EN, or CCX282B)—originally intended for patients with moderate-to-severe Crohn’s disease—has completed one Phase 2 trial in 67 patients with celiac disease [35]; however, despite the completion of the trial several years ago, no results relating to celiac disease have been made public or published.

A patent search (see Appendix Table A1) revealed at that at least a dozen patents (including U.S. and European patents) were granted for potential celiac disease-related therapies and diagnostics in 2013 and 2014. These were assigned to organizations including Alvine Pharmaceuticals Inc., Curemark LLC, Medarex Inc., Aesku Diagnostics GmbH & Co. KG, Immco Diagnostics Inc., Alba Therapeutics Corp., Nestec SA, BTG International Ltd., ImmusanT Inc., DSM IP Assets BV, Sigma Tau Ind Farmaceuti, and various institutions, groups and individuals.

## Clinical trial endpoints

An endpoint is quite simply a measure believed to quantify the potential effect of the treatment or intervention under study. Effect is, of course, a term that can be broadly interpreted. The effect is articulated in the claims made on the official drug label and such claims need to be supported by the study results, which are quantified by the endpoints. The process of thinking in terms of a drug’s mechanism of action (what it could do in theory), what it does in practice (clinical effect and efficacy), how this is demonstrated (endpoints) and how this relates into drug labeling claims (what is on the label) is not linear; in fact, regulatory consultants often advise that the thinking should begin around appropriate endpoints, with the end (labeling claims) in mind. In fact, this approach has found its canonization in what is known as the target product profile (TPP). Although not much used in a formal way, it nevertheless reflects how regulators think: the TPP is organized according to the key sections in the drug label and links drug development activities to specific concepts intended for inclusion in the drug labeling [36].

Clinical trial endpoints are well established in many major disease areas where a fair number of registration trials have already been conducted and drugs approved for marketing. Emerging fields, such as celiac disease—where there is little experience and no approved products—often lack agreed endpoints. Consequently, both sponsors and regulators will need to come to a new agreement for each development program. Many small biotechnology companies lack in-house regulatory experience and much frustration could be avoided by understanding, at an early stage, some general principles concerning primary endpoints for registration trials. In the following, we will review important concepts.

For registration trials, the FDA requires ‘clinically meaningful endpoints’ defined as endpoints that are direct measures of how patients feel, function, and survive [37] unless a validated surrogate biomarker acceptable to the FDA is available, which is rare. In disease in which there is a large subjective component, co-primary endpoints are increasingly employed: typically, a patient-reported outcome (PRO) is combined with an instrument that reflects disease activity more directly—for example, a biomarker. This approach is currently being implemented for Crohn’s disease and ulcerative colitis. Although the inflammatory bowel diseases have little in common with celiac disease, they are still similar with regard to ‘treatment success’ which would be incompletely captured if one were to focus on a single endpoint; for example histology or how the patient feels, but not both.

In celiac disease, conceptually, one type of treatment could control symptoms and prevent worsening of damage while another is, at least initially, focused primarily on healing and maintenance of healing, with little effect on symptoms. Obviously, in both cases, different endpoints or endpoint instruments are needed.

Several other food- and allergy-related disorders share a lack of well-defined clinical endpoints. An example is eosinophilic esophagitis, which occurs in response to an as-yet unknown allergen in the diet, has histological manifestations, has histology that does not correlate well with symptoms, and in which there is a requirement for an endpoint instrument that combines a PRO with objective response criteria [38].

Co-primary endpoints require that, to be an overall responder, patients meet the responder definitions for each of the individual endpoints that comprise the co-primary endpoint (see Glossary). Composite endpoints, in contrast, are endpoints that are composed of different measurements (e.g. several different scales, instruments or components) that are aggregated to an overall endpoint (see Glossary).

### Potential celiac disease endpoints

Categories of endpoints for consideration include the following (see Glossary for detailed definitions):

#### Patient-reported outcomes

In some diseases, PROs are more important than in others; compare, for example, depression with hypertension. A PRO is needed as either a primary endpoint or a component of a primary endpoint if the PRO alone is not sufficient to characterize improvements in how the patient feels, functions and survives.

We are aware of three published celiac-specific PRO questionnaires: Dorn 2010 (25 citations) [39], Leffler 2009 (18 citations) [40], and Häuser 2007 (36 citations) [41]. Given its recent publication, the CD-QoL by Dorn *et al.* has probably attracted the most attention. More generic questionnaires, such as the Gastrointestinal Symptoms Rating Scale (GSRS), have also been applied to measure celiac disease-related symptoms. Earlier work criticized the fact that, in celiac disease, the full dynamic range of the GSRS was not used [42]. Others have more recently demonstrated that the GSRS correlates with the ratio of the villus-height-to-crypt-depth—a promising histological outcome measurement instrument—as well as with laboratory test results, numbers of intraepithelial CD3+ cells, and serum levels of antibodies associated with celiac disease [43]. Regulatory authorities seem to have greater familiarity with the GSRS instrument than with others.

Celiac PROs are often described as having been validated, however, it is important to note that the FDA considers a ‘drug development tool’ (DDT) such as a PRO only ‘validated’ when it has undergone the FDA’s DDT qualification process [44]. There is currently only one instrument, the Exacerbations of Chronic Pulmonary Disease Tool for chronic obstructive pulmonary disease, which is validated through this relatively new procedure. The acceptability of any other PRO would have to be decided between the FDA and sponsor on a case-by-case basis.

A proprietary questionnaire being used by Alba in their Phase 2b multi-center trial ‘evaluating the efficacy, safety and tolerability of larazotide acetate in patients with celiac disease’ (see above) is of interest. Alba states that the questionnaire was developed according to the FDA’s Study Endpoints and Labeling Development (SEALD) guidelines, which is the FDA office involved in DDT qualification, and uses PROs [24].

A similar proprietary PRO, called the Celiac Disease Symptom Diary (CDSD), was developed by Alvine and published in abstract form in 2012 [45]. It collects data on symptoms common to celiac disease: abdominal pain, bloating, constipation, diarrhea, fatigue, flatulence, headache, nausea, skin rash, and problems with thinking clearly.

#### Histology

Histology instruments used to be considered the ‘gold standard’ for the diagnosis of celiac disease and, indeed, part of the definition of this disease; however, this has been challenged, and the European Society of Paediatric Gastroenterology, Hepatology and Nutrition (ESPGHAN) guidelines now consider HLA type and the results of serology to be of equal importance [15, 46]. The ESPGHAN criteria allow for the avoidance of biopsy in symptomatic children who have tTG IgA antibodies that are >10 times the upper limit of normal on an initial blood sample, followed by a confirmatory anti-endomysial antibodies (EMA) and HLA typing on a second blood draw. The response to a GFD can also be used to support the diagnosis. The approach outlined in the ESPGHAN guidelines has, however, not been endorsed by other societies with a predominantly adult focus such as the British Gastroenterological Society (BSG) or the American College of Gastroenterology (ACG). However, this approach has not been endorsed by adult societies, such as the British Gastroenterological Society (BSG) or the American College of Gastroenterology (ACG).

Although reasonable for diagnosis (in conjunction with other biomarkers), histology instruments are slow to respond to gluten-free diet, and complete normalization may take years. Another concern, currently unresolved, is the sometimes patchy distribution of disease activity, which can lead to sampling error. Also, there are various systems: Marsh, Marsh modified (Oberhuber), Corazza & Villanaci, and ‘morphometry’ (Table 1) [47–52].

**Table 1.** Histology scoring systems for celiac disease

	Publication	Focus	Scoring system
Marsh	Marsh and Crowe, 1995 [47], Marsh, 1992 [48]	Small intestinal mucosal immunopathology	Concludes that distinctive mucosal patterns typify experimental cell-mediated (T lymphocyte) reactions in small intestinal mucosa: the Type 1 (‘infiltrative’) lesion, Type 2 (‘hyperplastic’) lesion and the Type 3 (‘destructive’) lesion.
Marsh modified (Oberhuber)	Oberhuber *et al.,* 1999 [49]	A modified version of Marsh, revised with subcategories	Subcategorized Type 3 lesions based on villous height as Type 3A mild atrophy, Type 3B marked atrophy and Type 3C total villous atrophy.
Corazza & Villanaci	Corazza and Villanacci, 2005 [50]	A simpler grading system than Marsh, intended to minimize inter-observer disagreement	Grade A: Non-atrophic, with normal crypt and villus architecture and increased IELs (>25 IELs per 100 enterocytes). Grade B1: Atrophic, with villus-to-crypt ratio <3:1, but villi are still detectable and IELs are increased (>25 IELs per 100 enterocytes). Grade B2: Atrophic and flat, villi are not detectable and increased IELs are noted (>25 IELs per 100 enterocytes).
Ensari	Ensari, 2012 [51]	Author argues that duodenal biopsies have almost entirely replaced capsule biopsies of jejunal mucosa for the diagnosis of celiac disease and that the histological scoring systems need to be updated accordingly	Type 1: Normal villi with IELosis. Corresponds to Marsh Type 1, also present in the Oberhuber classification, and to grade A in Corazza &Villanaci’s proposal. Type 2: Shortened villi (<3:1 or <2:1 duodenal bulb) with IELosis and crypt hyperplasia. Corresponds to Types 3A and 3B in the Oberhuber classification and to grade B1 in Corazza & Villanacci’s proposal. Type 3: Completely flat mucosa with IELosis and crypt hyperplasia; corresponds to Marsh Type 3, and also to Type 3C in the Oberhuber classification, and to grade B2 in Corazza & Villanacci’s proposal.
Morphometry (Taavela)	Taavela *et al.,* 2013 [52]	The authors argue that, in contrast to scoring systems, histological morphometry provides continuous data that may be advantageous in practice and for clinical studies.	The tool is based on the quantitative morphological (villus-height-to-crypt-depth ratio; VH:CrD) and inflammatory (density of IEL) variables. Specimens must be rigorously assessed for optimal orientation.

IEL = intra-epithelial lymphocyte.

The known histology outcome instruments have limited inter-observer reliability and it is unclear whether the addition of immunohistochemical staining for intra-epithelial lymphocytes is always helpful (except in cases of suspected enteropathy-associated T-cell lymphoma) [53]. The context in which these histology instruments are used also needs to be considered; for example, the task of measuring histological deterioration—or lack of deterioration—in response to a gluten challenge in healed patients is easier than the determination of histological improvement after institution of a GFD. In the first scenario, histological changes may be evident as early as two weeks of after initiation of the gluten challenge [54]. As a result, histological distinction between patients treated with an active agent or placebo might also be expected to occur within that timeframe, with additional distinction over the following weeks.

Regardless of which histological outcome instrument is chosen for a primary analysis, morphometric data, such as villus-height-to-crypt-depth ratio [52], should probably also be collected for exploratory and sensitivity analysis. To optimize accuracy, the histological evaluation should take advantage of unbiased central reading with two independent readers. Cost-effective and scientifically rigorous approaches would include the use of central reading, proper collection of the biopsy material by the site endoscopist (location and number) and accurate specimen handling [55, 56].

#### Serology

Serological biomarkers are commonly used for the diagnosis of celiac disease in individuals who have not adhered to a gluten-free diet. Once patients do adhere to such a diet, the biomarker levels slowly decrease. Single measurements of celiac serologies, including serum anti-tTG IgA and anti-deamidated gliadin peptide (DGP) IgG antibodies, are generally viewed as not being particularly useful in assessing compliance with a gluten-free diet; however increases over time, especially in the context of a standardized gluten challenge, could be much more informative. There are currently limited data on their kinetics following a gluten challenge and any increase in serological markers may be delayed, compared with changes in histology [57].

#### Other tests

Intestinal permeability may be higher than normal in celiac disease patients [58], and quantified by the urinary lactulose/mannitol (LAMA) fractional excretion ratio. This test may be particularly relevant to drugs such as Alba’s larazotide acetate, a tight junction regulator; however, LAMA fractional excretion is very variable from person-to-person and could not differentiate between larazotide and placebo in a recent trial [27]. The authors suggested that differences in the method of collectingon of urine, compared with the method used in a previous proof-of-concept (POC) study [59], may have been relevant.

As mentioned earlier, the presence of gluten-reactive T cells in celiac disease can be detected using a cytokine release assay, following either a standardized gluten challenge or a challenge with principal gluten peptides, with any correlation to histological changes currently unknown [14].

#### Gene expression

Genetic biomarkers, specifically expression changes in the intestinal or duodenal mucosa, may be useful early biomarkers and expression changes in genes involved in regulating tight junctions and other adhesion molecules show promise [60].

### Endpoints used in recent clinical trials

We conducted a systematic review of all clinical trials listed on the World Health Organisation (WHO) International Clinical Trials Registry Platform [61], which collates information from clinicaltrials.gov and other regional and national clinical trials registries, using the search terms “celiac” and “coeliac”. We identified all celiac disease drug trials with endpoint information. The results are given in summary form by closely related or identical endpoints with trial numbers and URLs pointing to the trial where the respective endpoint is used. None of the trials are currently in Phase 3; most are at Phase 2 and a few Phase 1 trials have also explored efficacy endpoints (Table 2).

**Table 2.** Endpoints employed in recent celiac disease clinical drug trials

Endpoint	Entry in Clinical Trial Registry
Intestinal permeability	http://clinicaltrials.gov/show/NCT00362856 http://clinicaltrials.gov/show/NCT00386165 http://clinicaltrials.gov/show/NCT01257620
Clinical index of celiac disease activity	http://clinicaltrials.gov/show/NCT00492960 http://clinicaltrials.gov/show/NCT00889473 http://clinicaltrials.gov/show/NCT00859391 http://clinicaltrials.gov/show/NCT01396213 http://clinicaltrials.gov/show/NCT01765647
Villus-height-to-crypt-depth ratio	http://clinicaltrials.gov/show/NCT00540657 http://clinicaltrials.gov/show/NCT00620451 http://clinicaltrials.gov/show/NCT01661933 https://www.clinicaltrialsregister.eu/ctr-search/search?query=eudract_number:2013-003660-31 https://www.clinicaltrialsregister.eu/ctr-search/search?query=eudract_number:2010-023127-23 https://www.clinicaltrialsregister.eu/ctr-search/search?query=eudract_number:2009-012221-10 https://www.clinicaltrialsregister.eu/ctr-search/search?query=eudract_number:2007-003450-28 http://clinicaltrials.gov/show/NCT00962182 http://clinicaltrials.gov/show/NCT01917630
Histological scoring or not further specified	http://clinicaltrials.gov/show/NCT00671138 http://clinicaltrials.gov/show/NCT00959114 http://www.anzctr.org.au/ACTRN12609000738224.aspx http://www.trialregister.nl/trialreg/admin/rctview.asp?TC=1281
Changes in serology/biomarkers	http://clinicaltrials.gov/show/NCT00962182 http://www.anzctr.org.au/ACTRN12613001331729.aspx
Gluten concentration	http://clinicaltrials.gov/show/NCT02060864

### Other protocol considerations

Other considerations in protocol design include:

1. **Duration of gluten-free diet:** if the trial design includes a gluten challenge, significant mucosal healing must have occurred first. Requiring a GFD for periods of less than six months may be a risky strategy. There are two reasons for this: patients may not have learnt how to adhere to a GFD and one month may not be long enough to effect any changes. Trial participants should not be newly diagnosed, but should already have learnt how to adhere to a gluten-free diet over 6–12 months.
2. **Compliance with GFD**: a standardized interview with a skilled dietician is widely recommended; however, a recent pilot study of about 400 patients did not show an association between involving a dietician and severity of symptoms, adherence, or quality of life [62].
3. **Placebo arm**: a protocol with a placebo arm can be the best way to demonstrate a treatment effect, if one exists. In celiac disease, this would be unlikely to represent an ethical issue, at least in adults, since the illness is not life-threatening, usually does not cause major pain, and does not affect development. In pediatric patients, however, nutritional deficiencies may be more serious and a placebo arm might, depending on the length of the study, not be appropriate. One approach might be to have a gluten-free arm and one where gluten is re-introduced along with the investigational therapy.
4. **Baseline endoscopy:** The timing of this step needs to be considered carefully in view of the different time dynamics of changes in the biomarkers obtained (e.g. histology *vs.* expression analysis). Endoscopy is also important for exclusion patients who still have significant disease activity after having been on a GFD.

## Recommendations for endpoints in clinical trials of drugs for celiac disease

Phase 2 POC trials offer more flexibility on outcome measures, needing only to provide stakeholders with evidence to make a go/no-go decision. Ideally, this outcome instrument should also inform the development of potential endpoints in Phase 3 trials. To be truly useful, even in the POC stage, POC endpoints need to be able to reasonably predict ‘clinical benefit’, that is, in practical terms, correlate with outcome instruments acceptable for future registration trials. POC endpoints also need to be statistically efficient, i.e. take advantage of as much information as the data contain and be suitable for the most powerful hypothesis tests. Typically, this would favor continuous data over ordinal data or scores.

### Phase 2 proof-of-concept

Here, the primary endpoint should focus on objective outcomes that can be measured on a continuous scale—such as histological morphometry—rather than as a subjective histology score. It would be worth exploring the potential of newer serological biomarkers—including auto-antibodies, IgA anti-tTG, IgA anti-EMA, IgA anti-DGP, and REG 1α—along with gene expression in the gut mucosa [63]. Serial measurements of transthyretin (prealbumin), a protein reduced in malabsorption, were reported as an indicator of mucosal recovery in celiac disease in 2001 [64], and may be used as a non-invasive test but further validation of this approach has, to our knowledge, not been pursued. REG 1α, a molecule involved in the regeneration after autoimmune insults could, according to Vives-Pi *et al*.** [63], reflect mucosal recovery and could be useful as a serum biomarker. These authors state that changes in its concentration arise quickly and can be detected before specific autoantibodies are produced after the adaptive immune response has occurred.

Other (exploratory) endpoints should be chosen in Phase 2 according to their ability to inform the design of Phase 3 trials. Engagement of regulatory authorities is important, even at this stage.

### Phase 3 registration trials

Sponsors should consult with the relevant regulatory authorities long before the end of Phase 2 trials to gain insights into—and perhaps agreement on—appropriate primary endpoints for a Phase 3 trial. A PRO instrument will probably be required, in conjunction with a more ‘objective’ biomarker, probably histology. The biomarker could be a component of a co-primary endpoint together with the PRO, or perhaps a first-ranked secondary endpoint with a PRO instrument being the primary endpoint.

Specifically, the ratio of the villus height to crypt depth could be a suitable biomarker because of its continuous nature. This metric seems to be less subject to inter-observer variation and statistically more informative than other scoring systems. Even if a seemingly simple metric such as villus-to-crypt ratio is chosen, the reading process must be carefully standardized, for example, as described by Taavela *et al.* [52]. Another issue that should concern drug developers and regulators alike is mucosal healing. Complete mucosal healing is increasingly considered to be a pre-requisite for improved long-term outcomes in inflammatory bowel disease (as opposed to mere symptom control) [65] and there is parallel evidence in celiac disease that non-healing, i.e. persistence of villous atrophy, correlates with a higher risk of lymphoproliferative malignancy [66]. When patients with celiac disease can tolerate gluten intake thanks to drugs that keep symptoms at bay—perhaps because inflammatory injury and repair mechanisms are kept in balance—should we not still need to worry about long-term consequences? An ideal agent would help control symptoms and prevent acceleration of inflammation.

The choice of the right PRO instrument is a more difficult problem and experience in drug trials is limited. The greatest body of experience in this context has been accumulated through patients in Alba Therapeutics’ Phase 2b trial (342 patients, see above) where the “celiac disease domains of the gastrointestinal symptoms rating scale” were used [25].

A review of the FDA’s approach to trials in similar diseases with objective manifestations that have a large subjective component—including eosinophilic esophagitis and inflammatory bowel disease—could be useful in framing the discussion of endpoints for celiac disease drug trials. For example, Fiorentino *et al*. provide a scheme for an evolving partnership model for rational drug development (Table 3) [38].

**Table 3.** A partnership model for rational drug development to treat eosinophilic esophagitis (adapted from Fiorentino *et al.* [38]): a framework for similar trials in celiac disease?

Define EoE	Assess EoE natural history	Identify EoE assessment tools
Unify diagnostic criteria Use symptomatic and histological criteria	FDA and academia collaboration Pool multiple patient registries	Address the importance of EoE-specific COAs Raise questions on using general terms, such as dysphagia Identify the need for different COAs for pediatric and adult patients

Invite all stakeholders • Discuss overall plan	Standardize data entry • Interpret data from different sources	Evaluate intra-epithelial mucosal eosinophilia as a biomarker

Identify key issues Lack of well-defined and reliable COA	Recognize EoE subpopulation Define differences between pediatric and adult patients	

COA = clinical outcome assessment; EoE = eosinophilic esophagitis; FDA: U.S. Food and Drug Administration.

For eosinophilic esophagitis, clinicians and investigators are still debating the most appropriate clinical endpoints to define therapeutic response [67]; the situation in celiac disease will be no different but drug development still needs to continue, even in the absence of complete agreement.

## Conclusion

Research into celiac disease has entered an exciting phase: for the first time there may be alternatives to the gluten-free diet. As drugs move out of the proof-of-concept stage to Phase 3 confirmatory trials, they need to be evaluated by endpoints that are tailored to the drug, disease, and target patient population. Perhaps most importantly, these endpoints need to be acceptable to regulatory agencies. We have attempted to open a discussion of the issues that we find are relevant and important, which we hope academic researchers, industry and regulators will continue.

*Conflict of interest statement:* Drs. Gottlieb, Dawson and Hussain work for Quintiles, a company that provides bio-pharmaceutical development services and consulting. They have no other relevant disclosures.

 Joseph A. Murray has consulting arrangements with AMAG Pharmaceuticals, Entera Health, Inc, Sonomaceuticals, LLC, BioLineRx and is on the Alvine Pharmaceuticals, Inc. advisory board. Dr. Murray has received grants or research support from Alvine Pharmaceuticals, Inc., and Alba Therapeutics.

## Appendix

### Glossary

#### Biomarker

A physiological, pathological, or anatomical patient characteristic that is measured by an automated process or algorithm as an indicator of normal biological processes, pathological processes, or biological responses to a therapeutic intervention .^1^Composite endpoint.^2^

A single measure of effect, based on a combination of individual endpoints. These may combine patient-, observer- and clinician-reported outcomes with biomarkers. Particularly useful for drugs that can benefit patients in several ways or if component events are infrequent. Examples: cardiovascular death or hospitalization for heart failure; major adverse cardiac events (MACE): cardiovascular death, non-fatal myocardial infarction (MI), and non-fatal stroke; “clinical worsening,” may include categorical decline in functioning, worsening symptoms, addition of a new medication; hospitalization due to the disease, death, etc. (HRQOL instruments); often analysed as time to first event, or number of events over the study period.

#### Co-primary endpoint

Here, a clinical trial has multiple primary endpoints and the intervention is judged to have been effective only if it improves on *all* of the endpoints. In the case where an intervention is deemed effective if it improves on *at least one* of multiple endpoints, these are termed ‘alternative primary endpoints’.^3^ The challenge of co-primary endpoints was also discussed by Chuang-Stein *et al.* (2007).^4^

##### Effectiveness

An essential component of the basis for marketing approval of a drug: drugs must be safe and effective to justify approval. Effectiveness is defined as a benefit to patients in how they feel, function, or survive due to treatment with the drug.^5^

##### Endpoint

The way an assessment will be used as a study result and statistically compared between treatment groups to assess the effect of treatment. Endpoints are often named by the assessment measured, but a complete statement of the endpoint should include a full description of what data are collected and how they are analysed to support a specific study objective.^6^

##### First-ranked secondary endpoint

There is usually only one primary endpoint; if there are more than one, they are linked as co-primary endpoints with a logical AND. Very rarely alternative primary endpoints are used (logical OR). The next endpoint in the trial design hierarchy is the first-ranked secondary endpoint. Its principal role is to lend support to the primary endpoint. If both are met, results are considered to be more robust than if only the primary endpoint is positive. Sponsors will typically argue that co-primary endpoints are hard to meet and suggest that a co-primary dismantled into one primary and the first-ranked secondary endpoint. This approach allows for the trial to be successful, even if only the primary endpoint was reached. If the first-ranked secondary was not quite met but also pointed in the right direction, chances for regulatory approval are still high^7^.

##### Patient-reported outcome (PRO)

A PRO is a measurement based on a report that comes from the patient (i.e. study subject) about the status of a patient’s health condition without amendment or interpretation of the patient’s report by a clinician or anyone else. A PRO can be measured by self-report or by interview, provided that the interviewer records only the patient’s response. Symptoms or other unobservable concepts known only to the patient (e.g. pain severity or nausea) can be measured only by PRO measures. PROs can also assess the patient’s perspective on functioning or activities that may also be observable by others.^8^

##### Primary endpoint

All drugs have safety risks; therefore, the only reason that a patient would want to take a drug would be if the drug improved survival, resulted in a benefit that was detectable by the patient (improvement in symptoms, improvement in functional capacity), or decreased the chances of developing a condition or disease complication that is itself apparent to the patient and is undesirable (e.g. stroke). Therefore, a primary endpoint should be a direct measure of one of these. A primary endpoint should generally not be a measure of something that is not important to the patient (exception: validated surrogate endpoint).^9^

##### Secondary endpoint

Results that are measured at the end of a study, in addition to the main result (primary endpoint), to see if a given treatment worked. Secondary endpoints can explore other aspects of the treatment.^10^

##### Surrogate endpoint

A surrogate endpoint is a laboratory measure or a physical sign that is intended to be used as a substitute for a clinically meaningful endpoint. Ideally, the surrogate should exist within the therapeutic pathway, between the drug and meaningful benefit, i.e. the drug results in the therapeutic benefit by virtue of its effect on the surrogate. Changes induced by a therapy on a surrogate endpoint are expected to reflect changes in a clinically meaningful endpoint.^11^

**Table A1.** Results of an informal patent search, 2012–14

Patent title	Patent number	Abstract	Pub. date	Assignee
Peptides having protective effect towards the inflammatory activity of peptide 31–43 of a-gliadin in celiac disease	EP2758423 A2	Intended for preventive and therapeutic purpose by administration to subjects at high risk of developing celiac disease and/or celiac subjects just before a gluten containing meal is ingested.	July 30, 2014	Istituto Superiore di Sanità, CRA Consiglio per la Ricerca e la sperimentazione in Agricoltura
Methods and pharmaceutical compositions for treating celiac disease and gluten intolerance	EP2736525 A1	Oral administration of ALV003 may protect celiac disease patients and patients otherwise suffering from gluten intolerance from the harmful effects of ingesting food containing gluten.	June 4, 2014	Alvine Pharmaceuticals, Inc.
Treatment of celiac disease with IgA	US8709413 B2	Oral administration of an IgA or an IgM to the subject suffering from food allergy or food intolerance.	April 29, 2014	Michael R. Simon
Compositions and methods for treating celiac sprue disease	EP2718434 A2	This invention covers various polypeptides.	April 16, 2014	University of Washington, through its Center for Commercialization
Dietary management of celiac disease and food allergy	US20130344042 A1	Compositions and methods for dietary management of celiac disease and food allergy via enteral administration of at least one hydrolyzed protein and Lactobacillus rhamnosus GG (LGG).	Dec 26, 2013	Gretchen Tanbonliong
Methods of treating celiac disease	US20130323223 A1	Compositions which include digestive enzymes and which are formulated to reduce one or more symptoms of celiac disease or a related disorder.	Dec 5, 2013	Curemark, LLC
Compositions and methods for the therapy of inflammatory bowel disease	CA2522957 C	Examples for celiac and other diseases comprise anti-type 1 interferon antagonists, as well as polypeptides and small molecules that inhibit the interaction of Type 1 interferon with its receptor (IFNAR).	Oct 22, 2013	Medarex, Inc., Lesley B. Pickford, Christopher R. Bebbington, Geoffrey T. Yarranton, David King, Medarex, L.L.C.
Use of an immunologically reactive microbial transglutaminase for the diagnosis and/or therapy control of celiac disease or sprue	DE102012007510 A1	This involves microbial transglutaminase and its immunologically reactive portions or analogues, present in a complex with gliadin. A claim is also included for a diagnostic kit.	Oct 17, 2013	Aesku.Diagnostics GmbH & Co. KG
Methods and compositions for treating celiac disease	US20130266584 A1	The invention features the treatment of gastrointestinal disorders associated with an innate immune response triggered by alpha amylase inhibitor CM3, alpha amylase inhibitor 0.19 (0.19), CM1, CM2, CMa, CMd, CM16, CMb, CMX1/CMX3, CMX2, and/or alpha amylase inhibitor 0.53 (0.53).	Oct 10, 2013	Detlef Schuppan, Yvonne Junker, Towia Libermann, Simon T. Dillon
Compositions and methods for determining celiac disease	US20130109034 A1	The compositions include recombinant proteins that contain tissue transglutaminase and deamidated gliadin sequences. Also provided is a method to identify an individual as having celiac disease, based on the presence of antibodies.	May 2, 2013	IMMCO Diagnostics, Inc.
Method for treating celiac disease	US20130122086 A1	Enteric compositions comprising one or more tight junction agonists and/or antagonists are provided. Compositions may include a delayed-release coating.	May 16, 2013	Alba Therapeutics Corp.
Methods to predict risk for celiac disease by detecting anti-flagellin antibody levels	US8409819 B1	Methods, assays, and kits for predicting or stratifying the risk of celiac disease, based upon HLA-DQ genotype and/or anti-flagellin antibody levels.	April 2, 2013	Nestec S.A.
Methods and pharmaceutical compositions for treating celiac disease and gluten intolerance	WO2013016427 A1	Methods for protecting a subject from a deleterious effect of gluten ingestion, including oral administration of ALV003.	Jan 31, 2013	Alvine Pharmaceuticals, Inc. (applicant)
Treatment of celiac disease with IgA	US8313730 B2	A process for inhibiting symptoms of a subject with celiac disease is provided that includes administration of monoclonal-, or polyclonal-, monomeric, dimeric, or polymeric IgA.	Nov 20, 2012	Michael R. Simon
Diagnostic method and breath testing device	US20120234076 A1	A diagnostic method and breath-testing device for the diagnosis of celiac disease, using a hydrogen-selective sensor in the form of a ZnO nanowire-based sensor fabricated using a focused ion beam (FIB/SEM) instrument or a thin film.	Sept 20, 2012	Anastasia Rigas
New markers for the diagnosis of celiac disease	US20120225442 A1	New peptides and to their use in the diagnosis of celiac disease.	Sept 6, 2012	Karl Skriner
Epitopes related to coeliac disease	EP2486935 A1	Epitopes that are useful in methods of diagnosing, treating, and preventing coeliac disease and methods of using and detecting these epitopes.	Aug 15, 2012	BTG International Limited (applicant)
Compositions and methods for treatment of celiac disease	EP2367561 A4	Agents and vaccines for treating and diagnosing celiac disease. In particular, a combination of three peptides that are useful for treating and diagnosing celiac disease in a large proportion of patients.^1^	June 6, 2012	Immusant Inc
Testing efficacy for celiac disease	US20120107847 A1	A method to determine effectiveness of a compound or composition in treatment of celiac disease or gluten intolerance.	May 3, 2012	Dsm Ip Assets B.V.
Materials and methods for the treatment of celiac disease	US20120076861 A1	Materials and methods for the treatment of celiac disease and monitoring the treatment of such subjects.	March 29, 2012	University Of Maryland, Baltimore, Alba Therapeutics Corporation
Treatment of celiac disease with IgA	US8119104 B2	A process for inhibiting symptoms of celiac disease that includes administration of monoclonal-, or polyclonal-, monomeric, dimeric, or polymeric IgA.	Feb 21, 2012	Michael R. Simon

## Notes

1 http://www.fda.gov/Drugs/DevelopmentApprovalProcess/DrugDevelopmentToolsQualificationProgram/ucm370262.htm

2 http://www.fda.gov/downloads/Training/ClinicalInvestigatorTrainingCourse/UCM283378.pdf

3 Offen W, Chuang-Stein C, Dmitrienko A *et al.* Multiple Co-primary Endpoints: Medical and Statistical Solutions: A Report from the Multiple Endpoints Expert Team of the Pharmaceutical Research and Manufacturers of America. Therapeutic Innovation & Regulatory Science January 2007 vol. 41 no. 1 31–46.

4 Chuang-Stein C, Stryszak P, Dmitrienko A, Offen W (2007). Challenge of multiple co-primary endpoints: A new approach. Statistics in Medicine. 26, 1181–1192.

5 http://www.fda.gov/Drugs/DevelopmentApprovalProcess/DrugDevelopmentToolsQualificationProgram/ucm370262.htm

6 http://www.fda.gov/drugs/developmentapprovalprocess/drugdevelopmenttoolsqualificationprogram/ucm370262.htm

7 Authors’ definition and concept

8 http://www.fda.gov/drugs/developmentapprovalprocess/drugdevelopmenttoolsqualificationprogram/ucm370262.htm

9 http://www.fda.gov/downloads/Training/ClinicalInvestigatorTrainingCourse/UCM283378.pdf

10 EU Clinical Trials Register glossary: https://www.clinicaltrialsregister.eu/doc/EU_Clinical_Trials_Register_Glossary.pdf. Accessed on 13 October 2014.

11 http://www.fda.gov/downloads/Training/ClinicalInvestigatorTrainingCourse/UCM283378.pdf

## References

[1] Lammers K Vasagar B Fasano A Definition of celiac disease and gluten sensitivity. In: Rampertab SD Mullin GE (ed.). Celiac disease. New York: Springer, 2014, 13–25.

[2] Husby S Murray JA Diagnosing coeliac disease and the potential for serological markers. Nat Rev Gastroenterol Hepatol 2014;11:655–63. 25266110 10.1038/nrgastro.2014.162

[3] Green PHR Stavropoulos SN Panagi SG Characteristics of adult celiac disease in the USA: results of a national survey. Am J Gastroenterol 2001;96:126–31. 11197241 10.1111/j.1572-0241.2001.03462.x

[4] Ludvigsson JF Rubio-Tapia A van Dyke CT Increasing incidence of celiac disease in a North American population. Am J Gastroenterol 2013;108:818–24. 23511460 10.1038/ajg.2013.60 PMC3686116

[5] Fasano A Berti I Gerarduzzi T Prevalence of celiac disease in at-risk and not-at-risk groups in the United States: a large multicenter study. Arch Intern Med 2003;163:286–92. 12578508 10.1001/archinte.163.3.286

[6] Lohi S Mustalahti K Kaukinen K Increasing prevalence of coeliac disease over time. Aliment Pharmacol Ther 2007;26:1217–25. 17944736 10.1111/j.1365-2036.2007.03502.x

[7] Rubio-Tapia A Ludvigsson JF Brantner TL The prevalence of celiac disease in the United States. Am J Gastroenterol 2012;107:1538–44. 22850429 10.1038/ajg.2012.219

[8] Kaukinen K Lindfors K Mäki M Advances in the treatment of coeliac disease: an immunopathogenic perspective. Nat Rev Gastroenterol Hepatol 2014;11:36–44. 23917697 10.1038/nrgastro.2013.141

[9] Sapone A Bai JC Ciacci C Spectrum of gluten-related disorders: consensus on new nomenclature and classification. BMC Med 2012;10:13. 22313950 10.1186/1741-7015-10-13 PMC3292448

[10] FDA. Gluten and Food Labeling: FDA’s Regulation of “Gluten-Free” Claims [Internet]. 2013 [cited 2014 Nov 23]. Available from: http://www.fda.gov/food/resourcesforyou/consumers/ucm367654.Htm

[11] NPD Group. Food for thought: is gluten-free eating a trend worth noting? [Internet]. 2012 [cited 2014 Nov 23]. Available from: https://www.npd.com/perspectives/food-for-thought/gluten-free-2012.html

[12] Rubio-Tapia A Hill ID Kelly CP ACG clinical guidelines: diagnosis and management of celiac disease. Am J Gastroenterol 2013;108:656–76. 23609613 10.1038/ajg.2013.79 PMC3706994

[13] Coburn JA Voort JLV Lahr BD Human leukocyte antigen genetics and clinical features of self-treated patients on a gluten-free diet. J Clin Gastroenterol 2013;47:828–33. 23632357 10.1097/MCG.0b013e31828f531c PMC3735773

[14] Ontiveros N Tye-Din JA Hardy MY Ex-vivo whole blood secretion of interferon (IFN)-γ and IFN-γ-inducible protein-10 measured by enzyme-linked immunosorbent assay are as sensitive as IFN-γ enzyme-linked immunospot for the detection of gluten-reactive T cells in human leucocyte antigen (HLA)-DQ2·5+-associated coeliac disease. Clin Exp Immunol 2014;175:305–15. 24192268 10.1111/cei.12232 PMC3892421

[15] Husby S Koletzko S Korponay-Szabo I European Society for Pediatric Gastroenterology, Hepatology, and Nutrition guidelines for the diagnosis of coeliac disease. J Pediatr Gastroenterol Nutr 2012;54:136–60. 22197856 10.1097/MPG.0b013e31821a23d0

[16] Oxentenko AS Murray JA Celiac Disease: Ten Things That Every Gastroenterologist Should Know. Clin Gastroenterol Hepatol 2014 Jul 19. doi:10.1016/j.cgh.2014.07.024. [Epub ahead of print] 10.1016/j.cgh.2014.07.024 25051511

[17] Hall NJ Rubin GP Charnock A Intentional and inadvertent non-adherence in adult coeliac disease. A cross-sectional survey. Appetite 2013;68:56–62. 23623778 10.1016/j.appet.2013.04.016

[18] Hollon J Cureton P Martin M Trace gluten contamination may play a role in mucosal and clinical recovery in a subgroup of diet-adherent non-responsive celiac disease patients. BMC Gastroenterol 2013;13:40. 23448408 10.1186/1471-230X-13-40 PMC3598839

[19] Rubio-Tapia A Rahim MW See JA Mucosal recovery and mortality in adults with celiac disease after treatment with a gluten-free diet. Am J Gastroenterol 2010;105:1412–20. 20145607 10.1038/ajg.2010.10 PMC2881171

[20] Alvine Pharmaceuticals. Alvine Pharmaceuticals Doses First Patient in Phase 2B Trial for ALV003 in Celiac Disease [Internet]. 2013 [cited 2014 Nov 23]. Available from: http://www.alvinepharma.com/press-oct292013/

[21] Lähdeaho ML Kaukinen K Laurila K Glutenase ALV003 attenuates gluten-induced mucosal injury in patients with celiac disease. Gastroenterology 2014;146:1649–58. 24583059 10.1053/j.gastro.2014.02.031

[22] Tack GJ Van de Water JM Bruins MJ Consumption of gluten with gluten-degrading enzyme by celiac patients: a pilot-study. World J Gastroenterol 2013;19:5837–47. 24124328 10.3748/wjg.v19.i35.5837 PMC3793137

[23] Alba Therapeutics. Alba Therapeutics Announces Positive Results of Phase IIb Trial in Celiac Disease [Internet]. 2014 [cited 2014 Nov 23]. Available from: http://www.albatherapeutics.com/Portals/0/pdf/Alba%20Therapeutics%20FINAL%20Press%20Release%20CLIN1001-012%20Larazotide%20Acetate%20February%2011%202014.pdf

[24] Business Monitor News & Views. Alba gets exclusive rights to CeD PRO; recruiting for Phase IIb trial [Internet]. 2012 [cited 2014 Nov 27]. Available from: http://www.businessmonitor.com/news-and-views/alba-gets-exclusive-rights-to-ced-pro-recruiting-for-phase-iib-trial

[25] Wang C Rasmussen H Perrow W 929b Larazotide Acetate, a First In-Class, Novel Tight Junction Regulator, Meets Primary Endpoint and Significantly Reduces Signs and Symptoms of Celiac Disease in Patients on a Gluten-Free Diet: Results of a Multicenter, Randomized, Placebo Controlled Trial. Gastroenterology 2014;146(Suppl 1):S159.

[26] Leffler DA Kelly CP Abdallah HZ A randomized, double-blind study of larazotide acetate to prevent the activation of celiac disease during gluten challenge. Am J Gastroenterol 2012;107:1554–62. 22825365 10.1038/ajg.2012.211 PMC3463856

[27] Kelly CP Green PHR Murray JA Larazotide acetate in patients with coeliac disease undergoing a gluten challenge: a randomised placebo-controlled study. Aliment Pharmacol Ther 2013;37:252–62. 23163616 10.1111/apt.12147

[28] Immusan T. ImmusanT Initiates Clinical Trials of Nexvax2 Therapeutic Vaccine for Celiac Disease [Internet]. 2014. Available from: http://www.immusant.com/wp-content/uploads/2011/06/ImmusanT-P1b-FINAL-9.4.121.pdf

[29] Immusan T. ImmusanT’s Immunotherapy for Celiac Disease, Nexvax2, Selected as One of Informa’s Top 10 Autoimmune/Anti-Inflammatory Projects to Watch [Internet]. 2014 [cited 2014 Nov 23]. Available from: http://www.immusant.com/wp-content/uploads/2011/06/ImmusanT-TAP-Award-Press-Release-11.19.2014-FINAL.pdf

[30] Bioline RX. Drugs in Development: BL-7010 [Internet]. 2012 [cited 2014 Nov 23]. Available from: http://www.biolinerx.com/default.asp?pageid=14&itemid=20

[31] Clinicaltrials.gov. Safety and Systemic Exposure Study of BL-7010 in Well-Controlled Celiac Patients.[Internet]. 2014 [cited 2014 Nov 23]. Available from: https://clinicaltrials.gov/ct2/show/NCT01990885?term=BL-7010&rank=1

[32] Avaxia Biologics. Avaxia Biologics is Awarded Patent for its Proprietary Orally Active Antibody for Celiac Disease [Internet]. 2011 [cited 2014 Nov 23]. Available from: http://www.avaxiabiologics.com/docs/rev-11-12-13-Celiac-Disease-Treatment-Patent.pdf

[33] Acto GeniX. Clinical Stage Drug Development Platform Developing Therapeutics for GI, Mucosal and Immunological Diseases [Internet]. 2014 Sep [cited 2014 Nov 23]. Available from: http://www.actogenix.com/wp-content/uploads/Actogenix_Corporate_September-2014-Non-Confidential1.pdf

[34] Huibregtse IL Marietta EV Rashtak S Induction of antigen-specific tolerance by oral administration of Lactococcus lactis delivered immunodominant DQ8-restricted gliadin peptide in sensitized nonobese diabetic Abo Dq8 transgenic mice. J Immunol 2009;183:2390–6. 19635921 10.4049/jimmunol.0802891 PMC3480315

[35] ChemoCentryx. CCR9 Program [Internet]. 2014 [cited 2014 Nov 23]. Available from: http://www.chemocentryx.com/product/CCR9.html

[36] FDA. Guidance for Industry and Review Staff Target Product Profile — A Strategic Development Process Tool - Draft [Internet]. 2007 [cited 2014 Nov 23]. Available from: http://www.fda.gov/downloads/drugs/guidancecomplianceregulatoryinformation/guidances/ucm080593.pdf

[37] Fleming TR Powers JH Biomarkers and surrogate endpoints in clinical trials. Stat Med 2012;31:2973–84. 22711298 10.1002/sim.5403 PMC3551627

[38] Fiorentino R Liu G Pariser AR Cross-sector sponsorship of research in eosinophilic esophagitis: a collaborative model for rational drug development in rare diseases. J Allergy Clin Immunol 2012;130:613–16. 22857796 10.1016/j.jaci.2012.07.011

[39] Dorn SD Hernandez L Minaya MT The development and validation of a new coeliac disease quality of life survey (CD-QOL). Aliment Pharmacol Ther 2010;31:666–75. 20015103 10.1111/j.1365-2036.2009.04220.x

[40] Leffler DA Dennis M Edwards George J A validated disease-specific symptom index for adults with celiac disease. Clin Gastroenterol Hepatol 2009;7:1328–34.e3 19665584 10.1016/j.cgh.2009.07.031

[41] Hauser W Gold J Stallmach A Development and validation of the Celiac Disease questionnaire (CDQ), a disease-specific health-related quality of life measure for adult patients with celiac disease. J Clin Gastroenterol 2007;41:157–66. 17245214 10.1097/01.mcg.0000225516.05666.4e

[42] Revicki DA Wood M Wiklund I Reliability and validity of the gastrointestinal symptom rating scale in patients with gastroesophageal reflux disease. Qual Life Res 1997;7:75–83. 9481153 10.1023/a:1008841022998

[43] Taavela J Kurppa K Collin P Degree of damage to the small bowel and serum antibody titers correlate with clinical presentation of patients with celiac disease. Clin Gastroenterol Hepatol 2013;11:166–71.e1 23063678 10.1016/j.cgh.2012.09.030

[44] FDA. Drug Development Tools (DDT) Qualification Programs [Internet]. 2014 [cited 2014 Nov 27]. Available from: http://www.fda.gov/Drugs/DevelopmentApprovalProcess/DrugDevelopmentToolsQualificationProgram/

[45] Adelman D Leffler D Lebwohl B Celiac Disease Symptom Frequency and Severity Using A Disease-Specific Patient-Reported Outcome Diary: Observations From A Psychometric Validation Study in 202 Patients. Am J Gastroenterol 2012;107:S603.

[46] Husby S Murray JA New aspects of the diagnosis of celiac disease in children, adolescents, and adults. Mayo Clin Proc 2013;88:540–3. 23660481 10.1016/j.mayocp.2013.03.018

[47] Marsh MN Crowe PT Morphology of the mucosal lesion in gluten sensitivity. Baillieres Clin Gastroenterol 1995;9:273–93. 7549028 10.1016/0950-3528(95)90032-2

[48] Marsh MN Gluten, major histocompatibility complex, and the small intestine. A molecular and immunobiologic approach to the spectrum of gluten sensitivity (‘celiac sprue’). Gastroenterology 1992;102:330–54. 1727768

[49] Oberhuber G Granditsch G Vogelsang H The histopathology of coeliac disease: time for a standardized report scheme for pathologists. Eur J Gastroenterol Hepatol 1999;11:1185–94. 10524652 10.1097/00042737-199910000-00019

[50] Corazza G Villanacci V Coeliac disease. J Clin Pathol 2005;58:573–4. 15917404 10.1136/jcp.2004.023978 PMC1770677

[51] Ensari A Gluten-sensitive enteropathy (celiac disease): controversies in diagnosis and classification. Arch Pathol Lab Med 2010;134:826–36. 20524861 10.5858/134.6.826

[52] Taavela J Koskinen O Huhtala H Validation of morphometric analyses of small-intestinal biopsy readouts in celiac disease. PLoS ONE 2013;8:e76163. 24146832 10.1371/journal.pone.0076163 PMC3795762

[53] Järvinen TT Kaukinen K Laurila K Intraepithelial lymphocytes in celiac disease. Am J Gastroenterol 2003;98:1332–7. 12818278 10.1111/j.1572-0241.2003.07456.x

[54] Corazza GR Villanacci V Zambelli C Comparison of the inter-observer reproducibility with different histologic criteria used in celiac disease. Clin Gastroenterol Hepatol 2007;5:838–43. 17544877 10.1016/j.cgh.2007.03.019

[55] Lebwohl B Kapel RC Neugut AI Adherence to biopsy guidelines increases celiac disease diagnosis. Gastrointest Endosc 2011;74:103–9. 21601201 10.1016/j.gie.2011.03.1236 PMC3651876

[56] Evans KE Aziz I Cross SS A prospective study of duodenal bulb biopsy in newly diagnosed and established adult celiac disease. Am J Gastroenterol 2011;106:1837–42. 21606978 10.1038/ajg.2011.171

[57] Leffler DA Schuppan D Pallav K Kinetics of the histologic, serological and symptomatic responses to gluten challenge in adults with coeliac disease. Gut 2013;62:996–1004. 22619366 10.1136/gutjnl-2012-302196 PMC3525791

[58] Gopalakrishnan S Durai M Kitchens K Larazotide acetate regulates epithelial tight junctions in vitro and in vivo. Peptides 2012;35:86–94. 22401908 10.1016/j.peptides.2012.02.015

[59] Paterson B Lammers K Arrieta M The safety, tolerance, pharmacokinetic and pharmacodynamic effects of single doses of AT-1001 in coeliac disease subjects: a proof of concept study. Aliment Pharmacol Ther 2007;26:757–66. 17697209 10.1111/j.1365-2036.2007.03413.x

[60] Jauregi-Miguel A Fernandez-Jimenez N Irastorza I Alteration of tight junction gene expression in celiac disease. J Pediatr Gastroenterol 2014;58:762–7. 10.1097/MPG.0000000000000338 24552675

[61] World Health Organization. International Clinical Trials Registry Platform -Search Portal [Internet]. 2014 [cited 2014 Dec 4]. Available from: http://apps.who.int/trialsearch/

[62] Mahadev S Simpson S Lebwohl B Is dietitian use associated with celiac disease outcomes?. Nutrients 2013;5:1585–94. 23676548 10.3390/nu5051585 PMC3708338

[63] Vives-Pi M Takasawa S Pujol-Autonell I Biomarkers for diagnosis and monitoring of celiac disease. J Clin Gastroenterol 2013;47:308–13. 23388848 10.1097/MCG.0b013e31827874e3

[64] McMillan S Dickey W Douglas J Transthyretin values correlate with mucosal recovery in patients with coeliac disease taking a gluten-free diet. J Clin Pathol 2001;54:783–6. 11577127 10.1136/jcp.54.10.783 PMC1731281

[65] Neurath MF Travis SP Mucosal healing in inflammatory bowel diseases: a systematic review. Gut 2012;61:1619–35. 22842618 10.1136/gutjnl-2012-302830

[66] Lebwohl B Granath F Ekbom A Mucosal healing and risk for lymphoproliferative malignancy in celiac disease: a population-based cohort study. Ann Intern Med 2013;159:169–75. 23922062 10.7326/0003-4819-159-3-201308060-00006 PMC3788608

[67] Dellon ES Gonsalves N Hirano I ACG clinical guideline: evidenced based approach to the diagnosis and management of esophageal eosinophilia and eosinophilic esophagitis (EoE). Am J Gastroenterol 2013;108:679–92. 23567357 10.1038/ajg.2013.71

